# Going with the Flow: Sensorimotor Integration Along the Zebrafish GI Tract

**DOI:** 10.3390/cells14151170

**Published:** 2025-07-30

**Authors:** Millie E. Rogers, Lidia Garcia-Pradas, Simone A. Thom, Roberto A. Vazquez, Julia E. Dallman

**Affiliations:** Department of Biology, University of Miami, Coral Gables, FL 33146, USA; lxg1366@miami.edu (L.G.-P.); sxt1014@miami.edu (S.A.T.); rxv444@miami.edu (R.A.V.)

**Keywords:** zebrafish, autism, gastrointestinal, enteric nervous system, enteroendocrine cells, sensorimotor integration

## Abstract

Sensorimotor integration along the gastrointestinal (GI) tract is crucial for normal gut function yet remains poorly understood in the context of neurodevelopmental disorders (NDDs) such as autism spectrum disorder (ASD). The genetic tractability of zebrafish allows investigators to generate molecularly defined models that provide a means of studying the functional circuits of digestion in vivo. Optical transparency during development allows for the use of optogenetics and calcium imaging to elucidate the mechanisms underlying GI-related symptoms associated with ASD. The array of commonly reported symptoms implicates altered sensorimotor integration at various points along the GI tract, from the pharynx to the anus. We will examine the reflex arcs that facilitate swallowing, nutrient-sensing, absorption, peristalsis, and evacuation. The high level of conservation of these processes across vertebrates also enables us to explore potential therapeutic avenues to mitigate GI distress in ASD and other NDDs.

## 1. Introduction

**Figure 1:**
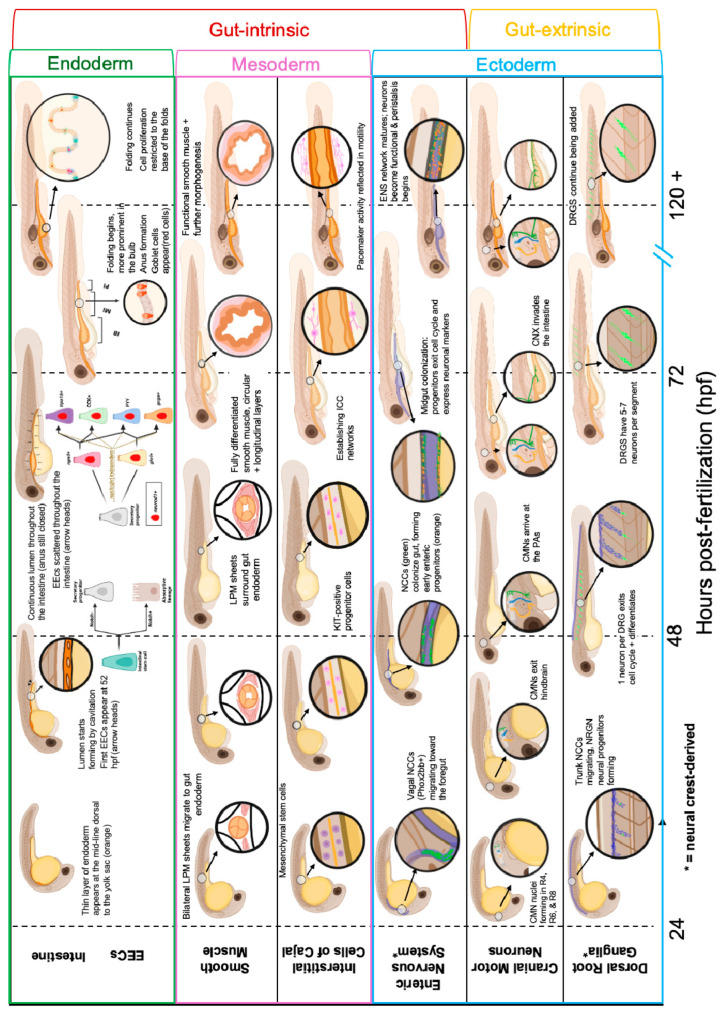
Developmental pathways of regulatory elements of gut sensorimotor reflexes. From top to bottom: intestinal epithelium/lumen, EECs, smooth muscle, ICCs, ENS, CMNs, and DRG. The timeline spans four developmental stages: 24 hpf, 48 hpf, 72 hpf, and 120+ hpf. Cell and tissue types are color-coded by germ layer (endoderm, green; mesoderm, pink; ectoderm blue) and avenue of regulation (gut-intrinsic, red; gut-extrinsic, yellow). Neural crest-derived tissues are indicated with an asterisk.

**Figure 2:**
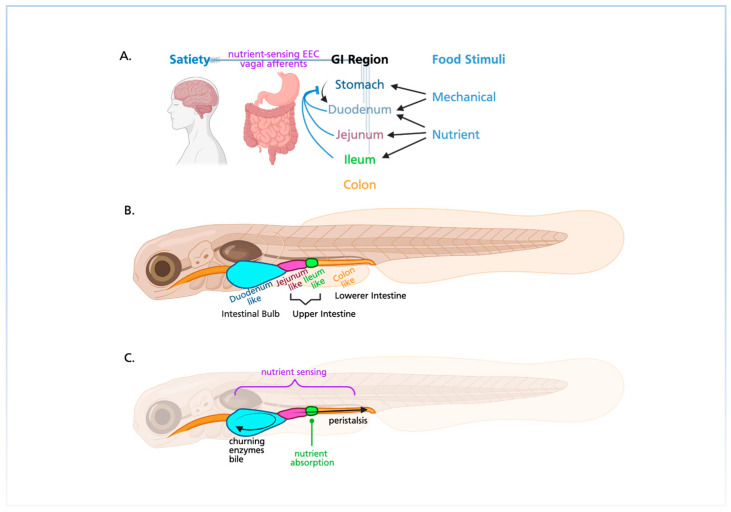
Digestive physiology in humans and the corresponding anatomical regions in zebrafish. (**A**) This diagram is adapted from [81] and shows how food stimuli activate EECs in the different regions of the gut. EECs release hormones that feedback on more anterior regions of the gut and promote satiety. (**B**) The regions of the zebrafish digestive tract and how they correspond to human GI regions. (**C**) Functional annotation of these regions includes arrows to indicate the propulsive force of smooth muscle contractions, an expansive region of nutrient sensing, and a compact ileum where peak nutrient absorption takes place.

Digestive function in vertebrates relies on the seamless integration of diverse, finely tuned sensorimotor circuits, made up of a multiplicity of regulatory elements that behave differently depending on whether the organism is hungry or satiated [1] (Figure 1 and Figure 2). Sensory inputs to the gut—such as mechanical stretch and nutrient signals—are detected and transformed into motor outputs and reflex arcs. Coordination among these circuits allows the gastrointestinal (GI) tract to maximize nutrient absorption when food is present, eliminate potential pathogens, and convert waste into bowel movements that are ultimately excreted. These various processes are integrated through feedback between the different regions of the GI tract and via communication with the central nervous system (Figure 2A).

When these finely tuned sensorimotor circuits are functionally disrupted in genetically defined neurodevelopmental disorders (NDDs), GI dysfunction can arise with significant clinical consequences. Autism spectrum disorder (ASD) presents a highly relevant example. ASD is a heterogeneous condition that affects more than 1% of the global population [2]. Individuals with ASD frequently experience GI dysfunction, with symptoms such as constipation, diarrhea, abdominal pain, and altered gut transit These symptoms are not only common, but often severe and persistent—exacerbating behavioral challenges and contributing to reduced quality of life [3,4,5].

Despite the high prevalence of digestive distress in ASD and other NDDs, the biological basis of these symptoms remains poorly understood [6,7]. Emerging evidence suggests that these GI symptoms may reflect intrinsic disruptions in the local circuits that regulate digestive physiology within the gut, as well as behavior through afferent and hormonal signaling to the brain. Many of the genes associated with ASD and GI distress (see Section 4) are expressed not only in the brain, but also in the enteric nervous system (ENS) and intestinal epithelium [8,9]. This pattern of co-expression hints at shared developmental vulnerabilities that may affect both gut and brain function.

A deeper understanding of how gut sensorimotor circuits are assembled and maintained in vertebrates is essential—not only for interpreting digestive symptoms, but also for advancing broader insight into the complex interplay between nervous systems, gut physiology, and environmental signals. To investigate the physiological consequences of these gene disruptions, researchers increasingly rely on in vivo models that recapitulate development and function of gut-regulatory circuits.

The zebrafish (*Danio rerio*) model offers a powerful platform to investigate how sensorimotor regulation of digestion emerges, how it breaks down in disease states, and how it might be targeted therapeutically [10]. Its optical transparency, external development, genetic tractability, and high degree of genetic conservation with mammals have enabled high-resolution studies of gut function from early development through larval and adult stages [10]. Many of these anatomical and functional aspects of the zebrafish intestine are homologous with the mammalian gut, making it a highly relevant model for studying vertebrate gut physiology [11,12].

Zebrafish models of various genetic conditions linked to ASD have been associated with convergent phenotypes such as impaired gut motility and reduced peristaltic contractions with deficits in cells that are part of sensorimotor circuits intrinsic to the gut [13,14]. These zebrafish findings are supported by work in the frog *Xenopus tropicalis*, which took a high-throughput, genetic knockdown approach to show altered maturation of enteric neurons is a convergent mechanism which could explain GI distress across five molecularly defined NDDs [15]. Taken together, these studies suggest that altered sensorimotor function within the gut may contribute directly to digestive distress in ASD and other NDDs.

This review synthesizes foundational and recent advances in our understanding of sensorimotor regulation along the zebrafish GI tract. We highlight how circuits in the pharynx, intestinal bulb, upper-, and lower-intestine contribute to key digestive behaviors and explore how various regulatory systems—including the vagus, ENS, enteroendocrine cells (EECs), and interstitial cells of Cajal (ICC)—interact to maintain function and homeostasis. We also discuss how disruptions to these systems in genetic models of NDDs affect gut function and offer insights into gut–brain communication more broadly. By integrating developmental and functional perspectives, we aim to provide a helpful resource for researchers exploring how gut regulation is affected in zebrafish and other vertebrate autism models.

## 2. Regulatory Elements of Gut Sensorimotor Reflexes

Here we focus primarily on a subset of regulatory elements of digestion, and only touch upon liver, pancreas, hypothalamus, etc. that merit deeper exploration beyond this review.

### 2.1. Gut-Extrinsic Regulators

#### 2.1.1. Cranial Motor Neurons

Here we focus on the development of the subset of cranial motor neurons (CMNs) that are involved in feeding and digestion, known as the branchiomotor neurons. These include the facial, or seventh cranial nerve (CNVII), the glossopharyngeal, or ninth cranial nerve, (CNIX), and the vagal, or tenth cranial nerve (CNX) [16,17]. CMN cell bodies are located in the brain and extend long axons that exit the brain ventrally on the left and right sides. Axons innervate intricate sets of muscles that produce head movements, including chewing and swallowing; other axons regulate the function of organs including the heart and intestine (Figure 1, 6th row).

Beginning at 16–24 h post fertilization (hpf), CNVII and CNIX exit the cell cycle and start to differentiate in the hindbrain rhombomeres (R) 4 and 6, respectively [18]. These newborn groups of neurons then migrate, CNVII toward R6 and R7 and CNIX towards R7 [18]. CNVII and CNIX then exit the brain and arrive at their target pharyngeal arches (PA), PA2 and PA3, respectively, by about 36 hpf [17,19]. CNVII and CNIX control the initial transportation of food through the mouth and pharynx [16,17]. Neurons of the CNX first exit the cell cycle by 24–36 hpf in R8, and then form a large, central fascicle consisting of axons poised to exit the hindbrain [19]. Hgf/Met signaling directs motor CNX axon targeting of the PAs, and by 34–54 hpf, four branches form and connect to PA4-7 [20]. The four branches innervating PA4-7 command oropharyngeal, pharyngeal, and some esophageal musculature, including those that enable swallowing and related processes. A fifth visceral branch then forms and extends caudally to innervate visceral organs, which includes the intestine [17,21]. At 52–72 hpf, CNX axons contact the gut between the proximal and medial intestine, at which point, fibers begin rapidly extending toward the distal intestine, while further innervation of the proximal intestine occurs more slowly. Overall, topography of CNX fibers is organized through Retinoic Acid signaling and is largely conserved between humans and zebrafish, enabling efficient investigation of CNX as it relates to gut motility [20]. By 5–7 dpf, bi-directional motor and sensory communication is functional and larvae start to feed with the CNX helping to regulate satiety and hunger.

#### 2.1.2. Dorsal Root Ganglia

The dorsal root ganglia (DRG) contain the cell bodies of sensory neurons that carry information from the periphery to the central nervous system [22] (Figure 1, 7th row). One of the tissues innervated by the DRG in mammals is the distal intestine [23]. DRGs develop in a rostral to caudal gradient, deriving from trunk neural crest that make their epithelial to mesenchymal transition in the dorsal neural tube at around 14 hpf [24,25]. The neural crest cells, marked by *sox10* expression, migrate ventrally down each trunk segment in streams, located between the neural tube and the somites [26]. One cell in each migrating stream of neural crest cells becomes a fate-restricted neuronal precursor marked by *neurogenin* expression; early neuronal differentiation is then marked by *brn1* and *isl1* expression and finally *runx1* or *runx3* expression in nociceptors and proprioceptors, respectively [22]. By 48 hpf, the first DRG neurons have extended neurites, and their survival and fate depend on growth factor signaling from the tissues they innervate [27]. The first wave of DRG neural crest differentiation gives rise to large diameter mechanosensitive and proprioceptive neurons, the second wave smaller diameter mechano-, thermo-, pain-sensitive neurons, and the third wave to glia. The specification of DRG at the expense of other neural crest fates involves wnt1 activation of nuclear beta catenin [22]. Unlike mammals and chick, zebrafish continue to make DRGs and sensory neurons as they mature; these new neurons arise from resident neural crest progenitors through a process that involves Notch signaling [28].

The role of the DRG in digestive function and distress is an emerging and important area across model systems. A recent study has shown that DRG neurons in mice innervate the distal intestine where they sense food-induced mechanical stretch, pain, and regulate immune responses [29]. Given that one of the primary symptoms in ASD-related GI distress is pain, the DRG presents a highly relevant target for direct therapeutic intervention.

### 2.2. Gut-Intrinsic Regulators

#### 2.2.1. Intestinal Epithelium/Lumen

Here we discuss the development of the intestinal epithelium, a thin layer of endoderm that begins forming at the midline, dorsal to the yolk, by 26 hpf (Figure 1, 1st row) [11,30]. A few hours later, the lumen begins forming rostrally in the esophageal region and the intestinal bulb primordium, progressing caudally through the gut tube. A continuous lumen occurs through the fusion of small cavities within the bilayered epithelium, which reorganizes into a simple columnar monolayer [11]. In contrast to mammals, zebrafish gut morphogenesis proceeds largely independently of apoptosis [11,31]. By 52 hpf, the gut tube extends from the prospective mouth to anus, and regional buds of the liver, pancreas, and swim bladder are apparent [11,30].

By 74–76 hpf, a continuous lumen spans the entire digestive tract—though the anus remains closed—and epithelial cells polarize, adopting a columnar morphology with basal nuclei [11]. Between 98 and 126 hpf, the intestine becomes fully functional, and three regions become distinguishable: the anterior intestinal bulb, the mid-intestine, and the posterior intestine [11]. The intestinal bulb replaces the mechanical, churning function of the acidic mammalian stomach, which is absent in fish [32]. Zebrafish also lack a cecum, which in mammals demarcates the transition between small and large intestines [11].

Until recently, it was believed that the digestive system’s lining consisted of mainly simple columnar epithelium. More recent studies, however, have identified a region of non-keratinized stratified squamous epithelium in the zebrafish upper digestive tract, which is remarkably similar to human esophageal epithelium [11,33]. This epithelial region, on the dorsal side of the digestive tract between the pharynx and intestinal bulb, is detectable at 5 dpf and at 7 dpf begins stratifying into four cell layers: the basal layer, parabasal layer, superficial layer, and dead cell layer. By 90 dpf, the epithelium is histologically similar to human esophageal epithelium. Adult zebrafish epithelia have papillae formed from multiple layers of parabasal and superficial cells, also seen in human esophageal epithelia. Basal transcription factors in human esophageal development, such as *p63*, *sox2*, and *pax9*, are also critically expressed in zebrafish larvae at 7 dpf.

Epithelial folding becomes evident starting at 98 hpf, particularly in the intestinal bulb, and, by 14 dpf, the intestine displays well-developed, irregular folds, rather than the finger-like villi characteristic of mammals [12]. Although zebrafish lack crypts of Lieberkühn, epithelial cell proliferation becomes restricted to the base of these folds by 5 dpf, functionally resembling the proliferative crypt zones found in mammals [11,12]. It has been shown that intestinal epithelial proliferation increases significantly in the presence of a normal microbiota, consistent with observations in mammals [34].

The intestinal epithelium includes multiple specialized cell types. Absorptive enterocytes are the most abundant and are identifiable by apical markers such as sodium phosphate cotransporter protein [12]. Goblet cells emerge around 100 hpf in the mid-intestine and are characterized by large, apical, mucin-containing theca [12]. EECs are first detected at 52 hpf in the posterior intestine, and, by 125 hpf, all of the various EECs subtypes are found in specific regions along the entire tract [11,35]. Although they comprise less than 1% of the intestinal epithelium, EECs collectively form the largest endocrine system in the body and play essential roles in digestion, appetite control, and systemic physiology [36,37]. For that reason, the differentiation and function of the EECs are described in greater detail in the section below.

#### 2.2.2. Enteroendocrine Cells

EECs arise from secretory progenitors within the intestinal epithelium, and although many aspects of EEC differentiation are conserved across species, there are significant differences between mammals and zebrafish (Figure 1, 2nd row). In zebrafish, *ascl1a*, expressed beginning around 36–40 hpf, is essential for the differentiation of all secretory cell types [38], while in mammals, this role is typically attributed to *atoh1* [39]. *ascl1a* acts upstream of a cascade that includes transcription factors such as *sox4b*, *neurod1*, *nkx2.2*, *rfx6*, *pax6b*, and *isl1*, expressed between 50 and 65 hpf [38,40]. Between 25 and 40 hpf, Notch signaling controls the balance between absorptive and secretory cell fates by repressing *ascl1a* expression in absorptive enterocytes during a critical developmental period [38]. In mammals, transient expression of the transcription factor *Neurogenin3* (*Ngn3*) directs progenitor cells toward an endocrine fate [41], and subsequent expression of *Neurod1* is required for further differentiation into mature EEC subtypes. In zebrafish, however, although *ngn3* is expressed in some EECs, it plays a more limited role. Instead, *neurod1* is both necessary and sufficient for EEC differentiation [35].

Mature EECs are typically classified based on the hormones they secrete, the size of the vesicles they contain, or their similarity to pancreatic cells, with significant advances in recent years [42]. In zebrafish, a study by Morash et al. [35] identified seven transcriptionally distinct clusters within the *neurod1*^+^ EEC population based on single cell RNA-sequencing analysis. A putative progenitor population was characterized by the absence of hormone gene expression and enriched for *ngn3*, *ascl1a*, and *sox4b*. In contrast, the remaining six clusters represented more differentiated EEC subtypes, each displaying unique combinations of hormone-encoding genes and transcription factors. Cluster 1 expressed high levels of the hormones *ghrl* (ghrelin), which regulates appetite and growth hormone release [43], and *sst2* (somatostatin), as well as transcription factors *insm1a* and *nkx2.2a*. Consistent with previous studies, this combination of genes indicates an early endocrine identity [11,44,45]. Cluster 2 was dominated by *pyyb* (peptide YYb), which is involved in regulating food intake [46], while cluster 3, positioned adjacent in pseudotime, expressed *gcga* (glucagon a). Cluster 4 was enriched for *insl5a*, *calca*, *gast*, and *nmbb*. Cluster 5 was defined by high expression of *ccka* (cholecystokinin a), a hormone known to regulate lipase and bile secretion [46]. Lastly, cluster 6 showed elevated expression of *tph1b*, which encodes the serotonin (5-HT) biosynthesis enzyme, along with *lmx1ba* and *lmx1al*, transcription factors known to drive serotonergic differentiation. These cells correspond to enterochromaffin-like cells, the primary source of peripheral 5-HT, producing approximately 90% of the body’s 5-HT [47]. 5-HT released from these cells regulates gut motility and secretion and influences satiety, visceral pain, and vagal signaling to the brain [48]. These different EEC subtypes emerge progressively between 3 and 6 dpf, following the initial appearance of EEC progenitors at around 52 hpf [11,35] and their distribution along the gut show a specific pattern related to their hormonal functionality. For instance, ghrelin-, peptide YY (PYY)-, and cholecystokinin (CCK)-secreting cells are located in the proximal region of the intestine, where nutrient digestion and absorption takes place; glucagon expression is found in the proximal and mid-intestine; and somatostatin- and 5-HT-producing EECs are present along the whole gut [35,46].

The functional maturation and physiological responsiveness of EECs are profoundly shaped by environmental factors, especially diet and the gut microbiota [49]. A recent study showed that, in conventionally raised larvae, microbial colonization promoted clustering of mitochondria at the basolateral side of EECs, where hormone exocytosis occurs, and nutrient-evoked calcium signaling was markedly increased in comparison to the germ-free animals, where mitochondria remain diffusely distributed [50]. When exposed to dietary fatty acids, only EECs from colonized larvae exhibited the expected calcium and ATP responses. These events are facilitated by the basolateral accumulation of mitochondria in mature EECs, allowing tight coupling between calcium fluxes and hormone vesicle release. Furthermore, indole, a microbial metabolite derived from tryptophan catabolism by bacteria like *Edwardsiella tarda*, can activate the EECs expressing the Trpa1 channel [49]. Once triggered, these cells release 5-HT, which stimulates intestinal motility and activates CNX sensory neurons. These Trpa1+ EECs have been observed to extend neuropod-like projections that form close contacts with vagal afferents [49]. These direct connections between EECs and neurons innervating the gut had been previously described in mice [51]. EECs are intimately connected to every aspect of the sensorimotor regulation of the gut, but they have a particularly close relationship with the ENS, covered in the next section. The proximity of EECs to enteric neurons along the intestine suggests that neuroepithelial communication may shape motility and absorption reflexes, but it remains unclear whether EECs and ENS influence one another with regard to maturation, circuit refinement, or activity-mediated plasticity during larval development.

#### 2.2.3. Enteric Nervous System

The ENS is an autonomous network of neurons and glia that innervates the digestive tract, facilitating critical functions such as nutrient absorption and intestinal motility [52] (Figure 1, 5th row). In mammals, the mature ENS is a network of thousands of interconnected ganglia embedded in the myenteric and submucosal plexuses of the gut [50]. By contrast, the zebrafish ENS is organized as a single myenteric plexus between the longitudinal and circular smooth muscle layers surrounding the lumen. There are only ~500 enteric neurons in the zebrafish gut, which do not form dense ganglia, making the zebrafish ENS readily accessible to whole-gut-level functional analyses [53].

The ENS derives primarily from vagal neural crest cells that delaminate from the neural tube and migrate ventrally from the hindbrain and then caudally in two parallel chains to populate the intestine [24,54]. The migration and proliferation of these enteric progenitors are tightly regulated by transcription factors and conserved signaling pathways. The RET/GDNF pathway plays a central role, where glial cell-derived neurotrophic factor (GDNF) binds to RET/GFRα receptor complexes (*gfra1a* and *gfra1b* in zebrafish) to promote enteric neural crest cell (ENCC) migration and survival [55,56,57]. The Hedgehog signaling pathway, particularly Sonic hedgehog, is also evolutionarily conserved and essential in ENS development. Disruptions in Sonic hedgehog signaling impair ENCC migration and gut colonization [58]. Semaphorin signaling, and in particular Sema3d is required for neural crest cell proliferation and directional migration during early hindbrain and gut development [59]. The homeobox gene *meis3* plays a key role in guiding ENCC migration [60]. Transcription factors including *sox10*, *phox2b*, *pax3*, and *foxd3* are also essential for proper ENS development. Knockdown studies have shown that loss of *sox10*, *phox2b*, or *pax3* results in the complete absence of ENS neurons [60]. Together, these signaling pathways and transcription factors interact in complex and coordinated ways to orchestrate the timing, direction, and fate of neural crest-derived progenitors during zebrafish gut colonization in a way that is conserved in mammals. The Endothelin pathway appears to play a non-essential modulatory role in ENS development in zebrafish. Unlike in mammals, where loss of Ednrb leads to severe ENS aganglionosis, mutations in *ednrb1* result in pigment cell defects but no obvious ENS migration or neural defects [60,61].

Across vertebrates, the ENS is modulated by inputs from the central nervous system (CNS), including vagal and DRG pathways. Vagal afferents project to the hypothalamus and dorsal vagal complex in the hindbrain—particularly the nucleus tractus solitarius (NTS)—where interoceptive signals and integrated and relayed to the dorsal motor nucleus of the vagus to coordinate autonomic output [62]. However, the core processes of digestion are regulated by intrinsic ENS circuits. The sensory intrinsic primary afferent neurons (IPANs) detect stretch and chemical signals even though they do not directly contact the intestinal lumen [53,63,64]. IPANs synapse onto interneurons in the myenteric plexus that integrate sensory input and coordinate activity across motor neurons. Excitatory motor neurons stimulate contraction of smooth muscle upstream of a luminal bolus, while inhibitory motor neurons induce downstream relaxation, facilitating forward propulsion of food through peristaltic motility [53,64,65]. Zebrafish possess at least 10 identified enteric neuron subtypes, many of which express conserved neurotransmitters including 5-HT, acetylcholine (ACh), nitric oxide (NO), vasoactive intestinal peptide (VIP), and pituitary adenylate cyclase-activating polypeptide [66,67,68].

These ENS neurons are spatially distributed and exhibit functional specialization in the regulation of peristalsis, segmental contractions, and secretion. The timing of differentiation during migration is subtype-specific, resulting in regionally enriched populations [69]. Nitrergic enteric neurons, the most abundant subtype, differentiate earliest (~56 hpf) and are distributed relatively evenly along the length of the gut. Serotonergic neurons, on the other hand, are enriched in the anterior segments of the gut and gradually decrease toward the distal intestine [67]. These spatial differences reflect the regionalization of different sensorimotor functions—the higher density of serotonergic neurons in the anterior gut suggests an increased capacity to initiate and regulate strong contractions that corresponds with the activity of the intestinal bulb. The widespread distribution of nitrergic neurons means that coordinated inhibitory reflexes critical to peristalsis can be activated in any region. These patterns of regional specialization likely arise from tightly regulated developmental programs. Recent advances in imaging and sequencing have enabled high-resolution tracking of how these spatial and functional distinctions emerge during ENS development.

Single cell sequencing and immunohistochemistry techniques have made it possible to quantify and visualize the developmental dynamics underlying spatiotemporal organization of enteric cell types. Zebrafish transgenic lines, such as Tg(*phox2bb*:Kaede), provide excellent tools for visualizing enteric progenitor proliferation [70]. This line uses UV-induced Kaede protein photoconversion to track cell division: red Kaede fluorescence dilutes with rapid cell proliferation, while newly synthesized green Kaede accumulates, allowing quantification of proliferation rates across developmental stages. Single-cell atlases have further mapped distinct neural crest-derived lineages, revealing the diverse transcriptomic profiles of enteric derivatives [71]. Despite these advanced tools and a growing body of research, several components of ENS integration remain to be understood—most notably, the interaction between enteric neurons and other non-neuronal partners such as smooth muscle cells, ICCs, glia, and EECs. While EEC subtypes and differentiation pathways have been characterized in zebrafish [35], and described above, the functional connectivity between EECs and the ENS is not yet well defined.

#### 2.2.4. Smooth Muscle

Smooth muscle in the zebrafish intestine plays an essential role in coordinating gut motility through propagating contractions (Figure 1, 3rd row). These muscles are organized into concentric layers—an inner circular and an outer longitudinal layer—mirroring the architecture of the mammalian gut [11]. Differentiation of smooth muscle begins during early larval development and is largely complete by 5 dpf, aligning with the emergence of functional peristalsis [72]. As in mammals, intestinal smooth muscle cells are derived from the lateral plate mesoderm, which approach the developing gut bilaterally and give rise to migratory smooth muscle progenitors, expressing key markers such as *hand2*, by 48–72 hpf. These progenitors surround the gut tube and, under the influence of TGFβ signaling, differentiate into a common smooth muscle precursor population. Sequential expression of other key markers, such as non-muscle myosin heavy chain (around 50 hpf), *sm22α-b* (by 56 hpf), and *acta2* (by 60 hpf), mark the commitment to the intestinal smooth muscle cell lineage and transition to mature smooth muscle. By 5 dpf, these cells organize into distinct circular and longitudinal muscle layers surrounding the gut lumen [12,72].

Between 3 and 5 dpf, functional neuromuscular junctions begin to form between differentiated muscle cells and the defined axonal projections of nearby differentiated enteric neurons [69]. The morphology and organization of the smooth muscle layers continues until 6 dpf, at which point the cells are fully differentiated. Confocal imaging of Tg(*sm22α-b*: GFP) larvae reveals that the cells in the inner circular smooth muscle layer directly surround the intestinal epithelium, while the cells of the longitudinal smooth muscle form above it [72]. While the ENS and ICCs serve as the primary coordinators of gut motility, smooth muscle contractility can also be modulated by extrinsic signals—for example, bile acids released by the liver can activate TGR5 receptors on enteric neurons and alter motility patterns, linking hepatic function to gut sensorimotor regulation [73].

A recent study by Okamoto et al. [74] employed calcium imaging in transgenic zebrafish larvae expressing *GCaMP3* under a smooth muscle-specific promoter to visualize activity in the circular muscle layer. They observed that calcium transients in smooth muscle cells were tightly coupled to motility patterns and that contractions originated predominantly from the oral side of luminal content, propagating either anterogradely or retrogradely. This revealed a fundamental role for smooth muscle in establishing directional flow [74]. Moreover, optogenetic stimulation using *channelrhodopsin-2* confirmed that targeted activation of smooth muscle cells was sufficient to induce localized contractions and influence intestinal transit; demonstrating that zebrafish smooth muscle not only develops early and independently of the ENS but actively participates in shaping gut motility from the onset of feeding. These findings highlight zebrafish as a powerful model to study the development, function, and plasticity of intestinal smooth muscle and its integration with neural and epithelial components of the sensorimotor reflex network.

#### 2.2.5. Interstitial Cells of Cajal

ICCs are specialized mesenchymal cells that act as pacemakers in the GI tract, generating slow-wave electrical activity that coordinates rhythmic smooth muscle contractions (Figure 1, 4th row). In mammals, ICCs also mediate communication between enteric neurons and smooth muscle, playing a critical role in regulating peristalsis, sphincter relaxation, and gut tone [75]. Although less extensively characterized, ICC-like cells have been identified in zebrafish and are thought to fulfill similar functions [76,77]. Live imaging studies in zebrafish have revealed rhythmic gut contractions that persist even in the absence of enteric neuronal input [78], supporting the hypothesis that ICCs serve as intrinsic pacemakers early in development. Their interaction with other gut components—particularly enteric neurons and smooth muscle—appears critical for full maturation of peristalsis and segmental coordination.

Immunohistochemistry and ultrastructural analysis have described a population of elongated, stellate-shaped cells in the zebrafish gut that exhibited close anatomical associations with enteric neurons and smooth muscle fibers—features consistent with ICC morphology. These cells were distributed throughout the intestinal musculature and showed dynamic changes during larval development [77]. Zebrafish ICC-like cells also express kit (the ortholog of mammalian *c-Kit*), a receptor tyrosine kinase that is a canonical marker and essential regulator of ICC differentiation and maintenance. Inhibition of kit signaling using pharmacological agents such as imatinib mesylate disrupts intestinal transit and smooth muscle coordination in zebrafish larvae, suggesting functional conservation of ICC roles across vertebrates [79].

Despite recent advances, the molecular identity and functional subtypes of zebrafish ICCs remain incompletely defined. Tools such as *kit*-reporter lines or single-cell RNA sequencing could clarify the heterogeneity and regulatory inputs of this cell population. Given their sensitivity to genetic and environmental perturbation, ICCs represent an important, yet underexplored, component of the zebrafish gut motility circuit.

## 3. Integration of Sensorimotor Reflex Circuits Along the GI Tract

Food-seeking initiates foraging behaviors and, if these are successful, consumption of food. This process is motivated by either hunger or pleasure when foods are especially appetitive [1,80]. The first steps are depositing the food into the mouth and swallowing, both of which require fine motor control that can be disrupted in people with NDDs [6]. Once swallowed, the food bolus is mechanically and enzymatically broken down in the stomachs of mammals or in the intestinal bulb of zebrafish to convert the food to nutrients that can be absorbed. Throughout digestion, sensorimotor circuits composed of ICCS, EECs, and ENS regulate intestinal smooth muscle contractions that propel food, secretions, and waste through the GI tract (Figure 2).

In addition to GI motility, this sensorimotor circuit also regulates many other GI reflexes, including nutrient-sensing, immune regulation, and segmentation [49,65,82,83]. Nutrient-sensing EECs and promotility ECs interact for optimal nutrient absorption. Stomach-emptying and intestinal motility in the anterior GI tract are regulated by nutrient-sensing in the lower GI tract. As such, discrete boluses of nutrients are served up to small intestine and, once nutrient absorption is complete, another round of stomach emptying and nutrient absorption resumes until all available nutrients are transferred to the body [81]. Evidence that similar pulsatile delivery of nutrients occurs in zebrafish is suggested by movies of fluorescent beads transiting the gut that are segmented as they enter the intestine, a process that is disrupted in the shank3 zebrafish autism model [13], but this is an understudied aspect of zebrafish gut physiology. Thus, these sensory endocrine cells coordinate gut behaviors in a way that is sensitive and appropriate to gut contents.

### 3.1. Swallowing Reflex

The swallowing reflex in zebrafish involves rhythmic jaw and pharyngeal movements—analogous to the swallowing reflex in mammals—that are mediated by branchiomeric motor circuit. In both humans and zebrafish, the pharyngeal and buccal musculature involved is striated and controlled by cranial nerves; most predominantly: the CNVII, CNIX, and CNX [84]. CNX plays a central role in regulating the swallowing reflex as well as in establishing sensorimotor communication between the digestive tract and CNS [85]. CNVII and CNIX supplement this function by innervating key muscle groups in the pharynx and esophagus [86].

Swallowing is classically divided into three sequential stages: oral, pharyngeal, and esophageal [87]. In zebrafish, the oral phase begins with branchiomeric and associated muscle groups coordinating a rapid expansion and closure of the buccal cavity, generating the negative pressure for suction feeding [88,89]. Muscles such as the sternohyoideus, protractor hyoideus, and levator arcus palatini enable suction, while closure of the cavity is mediated by the adductor mandibulae complex, adductor operculi, and hyohyoidei adductores muscles. This coordinated muscle activity enables zebrafish to draw in prey and other food items. The oral phase is followed by the pharyngeal phase, which is highly conserved across humans and zebrafish, and involves the sequential constriction of pharyngeal walls to propel the ingested material along the esophagus towards the intestinal bulb [90]. This is achieved by using surrounding branchiomeric muscles. The pharyngeal swallow reflex, albeit brief, is considered one of the most complex reflexes overseen by the CNS [91]. Despite one-third of its muscle being striated, the upper esophagus falls entirely under CNX modulation [92]. By contrast, lower esophageal motility depends on smooth muscle and the ENS [93]. The esophageal phase mobilizes the bolus through peristaltic movement, in which rhythmic contraction of smooth muscle pushes food items down the lower esophagus and into the intestinal bulb to mix and digest food for later absorption. These concerted movements also serve to prevent regurgitation.

### 3.2. Peristalsis and Luminal pH

Peristalsis is the rhythmic, wave-like movement that propels ingested material through the digestive tract. This intrinsic smooth muscle reflex circuit is driven by ICCs and regulated by the ENS, with critical inputs from the CNS to modulate motility in response to internal and external cues [75,94] Peristalsis relies on highly coordinated cycles of local and propagating contraction and relaxation along the length of the intestine. When stretch is detected by ECs and IPANs, excitatory interneurons propagate the signal upstream to excitatory motor neurons [49,65]. These motor neurons release acetyl choline into the neuromuscular junction, inducing the circular smooth muscle oral to the bolus to contract and push it forward. The longitudinal muscles caudal to the bolus contract to shorten the length of the gut segment and advance the bolus. Simultaneously, the original stretch input activates inhibitory interneurons, which stimulate inhibitory motor neurons to release nitric oxide. This induces relaxation in the circular smooth muscle anal to the bolus, allowing it to move into the relaxed segment [67]. This occurs throughout the zebrafish digestive tract, with region-specific patterns that reflect distinct functional roles. While the esophagus and upper intestine exhibit stereotyped, unidirectional waves that propel ingested material posteriorly, the intestinal bulb displays bidirectional mixing contractions, and the lower intestine engages in slower, segmental movements to regulate waste transit and microbial interactions [95,96]. Live imaging techniques, including optogenetics and calcium imaging, allow investigators to observe and manipulate the cells controlling these peristaltic waves as a bolus moves down the digestive tract in transgenic zebrafish larvae [49,50,74].

In addition to coordinating motility, the ENS controls luminal pH to create an optimal environment for digestive enzymes and nutrient solubilization, while also influencing the composition and function of the intestinal microbiota. EECs also play a critical role here, sensing changes in luminal pH and relaying these signals to the ENS, which can rapidly adjust secretory and motility patterns to optimize food breakdown [97].

### 3.3. Churning and Enzymatic Reflexes of the Intestinal Bulb

The zebrafish intestinal bulb, located posterior to the esophageal-intestinal junction, does not have a perfect human analog. In mammals, the small intestine follows the stomach and consists of three sequential regions: the duodenum, the jejunum, and the ileum (Figure 2A). Based on gene expression, the zebrafish intestinal bulb is analogous to the mammalian duodenum [98]. However, unlike the duodenum, the intestinal bulb is characterized by bidirectional and non-propagative contractions that provide mechanical disruption, allowing for transient storage and homogenization of swallowed contents [99]. Indeed, the intestinal bulb is the region with the largest luminal space along the zebrafish digestive tract [98]. ICCs act as pacemakers, while the ENS modulates their frequency and propagation. One example of this can be seen post feeding, when cholinergic enteric neurons release ACh to stimulate smooth muscle contractions and increase anally directed peristalsis [100]. Orally directed peristalsis in the intestinal bulb is regulated specifically by a population of enteric neurons that express hyperpolarization-activated cyclic nucleotide-gated potassium channel 4 (HCN4) [101].

The intestinal bulb also contains a population of EECs which detect luminal nutrients such as glucose, microbial metabolites, fats, and amino acids [11,46]. Nutrient stimulation causes membrane depolarization and large increases in intracellular calcium, triggering the release of hormones and/or neurotransmitters to coordinate digestive responses. For example, a subset of EECs secrete CCK, inducing the delivery of bile from the gall bladder and liver to emulsify fats [46,102]. Functionally, the intestinal bulb acts as a sensorimotor hub and relay station—integrating luminal signals from EECs and neural inputs from ENS and vagal efferents to coordinate digestion [83,103].

### 3.4. Nutrient Sensing and Absorptive Reflexes

Following the intestinal bulb is the upper intestine, analogous to the mammalian jejunum and ileum. While absorption occurs more broadly, the ileum is the site where most nutrients are absorbed. This region is marked in both baby mammals and fish by Lysosome Rich Enterocytes [98,104,105] (Figure 2). In zebrafish, the upper intestinal region is responsible for nutrient sensing, absorption, and anally directed transit via peristalsis.

In mammals, nutrient-sensing EECs in the upper intestine have been shown to play a prominent role in maximizing nutrients absorption by regulating CNS-mediated feeding behaviors and motility in upstream regions of the gut [81]. For example, peristalsis and gastric emptying can be temporarily inhibited until nutrient absorption is complete and motility resumes. This dynamic feedback mechanism optimizes nutrient acquisition before the arrival of the next bolus. These EECs also coordinate with accessory digestive organs, including the liver and pancreas, that help prepare nutrients for absorption. A subset of EECs known as neuropods directly activate vagal afferents via glutamate [51,83,106]. These vagal signals are relayed to integrative brain centers, including the NTS, secondary gustatory nucleus, and the hypothalamus. These hubs, among others, integrate endocrine and neural regulation of gut function to invoke satiety.

While these functional circuits have not been definitely identified in zebrafish, live-imaging of digestion has shown a pattern of motility that creates small boluses of food in the upper intestine consistent with regulation of motility in upstream gut regions [13]. Moreover, an afferent sensory connection has been established between EECs and vagal neuronal pathways in the context of catabolic tryptophan sensing [49].

### 3.5. Evacuation Reflexes

The lower intestine, encompassing the distal-most region of the zebrafish intestine, is responsible for the final stages of digestion and the evacuation of waste. Zebrafish and mammalian evacuation share functional similarities, including reliance on coordinated smooth muscle contraction and control by enteric circuits [107]. Motility in the lower intestine is characterized by strong, unidirectional peristaltic waves that drive compacted material toward the cloaca, suggesting that the hindgut operates under a distinct set of sensorimotor reflexes optimized for waste clearance [11].

Live imaging of pooping events reveals stereotyped motor sequences that suggest rhythmic control of cloacal opening and abdominal contraction—behaviors reminiscent of mammalian evacuation reflexes [108,109]. In zebrafish, enteric neurons expressing nitric oxide synthase and VIP are enriched in the posterior gut and may facilitate coordinated muscle relaxation to support evacuation [53,67,110]. EECs in the lower intestine have also been implicated in modulating evacuation. These cells can sense the chemical composition of retained waste and release neuromodulators such as 5-HT, which may stimulate local reflex circuits or trigger cloacal contractions via enteric pathways [111]. Environmental and microbial cues can also modulate evacuation. For example, overgrowth of *Vibrio* species in the zebrafish intestine has been shown to induce changes in motility and intestinal transit, particularly in the posterior region, to prevent bacterial overgrowth [96]. This highlights the responsiveness of evacuation reflexes to microbial composition and supports the broader role of the gut–brain-microbiota axis in regulating digestive behaviors.

In mammals, DRG sensory neurons innervate the lower intestine, where they detect mechanosensory stretch and visceral pain [112]. Zebrafish orthologs *piezo1*, *piezo2a*, and *piezo2b* have been identified and found to express similar genes to their mammalian counterparts, but their developmental and functional roles in the intestine remain an understudied area in zebrafish [113,114,115].

## 4. Altered Sensorimotor Integration in ASD: Insights from Zebrafish and Other Animal Models

Below we describe recent work in animal ASD/NDD models that have revealed gut-regulatory mechanisms that may explain the prevalence of GI distress in these conditions.

### 4.1. CHD7

De novo loss-of-function mutations of the chromodomain helicase DNA-binding protein 7 gene (*CHD7*) result in CHARGE syndrome. Clinical features of CHARGE include GI dysmotility, swallowing difficulty, and gastroesophageal reflux linked to dysfunction of CNVII, CNIX, and CNX [116,117]. Morphologically, *CHD7*-deficient zebrafish effectively recapitulate human CHARGE syndrome phenotypes, demonstrating various physiological symptoms such as pericardial edema, cardiomegaly, small eyes, auditory deficits, and more [118,119]. Such models have provided insight into related GI complications; one study observed swallowing difficulties, a shortened GI tract, underdeveloped enteric nerve cell bodies, and reduced CNX and ENS innervation throughout the digestive tract [119]. 

### 4.2. CHD8

CHD8 syndrome is caused by haploinsufficiency in the chromodomain helicase DNA-binding protein 8 gene (*CHD8*). Up to 80% of people with CHD8 syndrome report GI distress of some kind. This altered GI motility is recapitulated in zebrafish models, which exhibit altered gut development [14,120]. Reduced numbers of enteric neurons and ECs indicate that hyposerotonemia could contribute to GI distress in CHD8 syndrome [14]. *Chd8* knockdown in *Xenopus* shows reduced ENCC migration [15].

Recent efforts have identified a potential connection between *CHD7* and *CHD8* via a novel protein FAM124B [121]. Furthermore, within neural stem cells and the fetal brain, *CHD8* has been shown to target other genes linked to NDDs, including *SHANK3*, *DYRK1A*, and *SYNGAP1* [122]. 

### 4.3. CNTNAP2

Contactin-associated protein-like 2 (CASPR2) is a cell-adhesion molecule that facilitates inter-neuron communication, organizes myelinated neuron microdomains, and plays a central role in neural development [123]. Homozygous mutations in the *CNTNAP2* gene in humans result in significant neurodevelopmental impacts including an elevated ASD risk and GI distress among other symptoms [6,124,125]. Whole gut transit is slowed in mice models alongside shortened repetitive action of colonic musculature [126]. Zebrafish models containing a double loss-of-function mutation (*cntnap2a/b*) show altered behavioral phenotypes, however GI function has not yet been assessed [127,128].

### 4.4. DYRK1A

Dual-specificity tyrosine phosphorylation-regulated kinase 1A (*DYRK1A*) is a protein kinase that is critical for neural progenitor proliferation and neuronal differentiation in humans [129]. Mutations in DYRK1A result in a syndromic phenotype that can include ASD with intellectual disability and prevalent GI symptoms. *Xenopus tropicalis* revealed *dyrk1a* knockdown models showed disrupted ENCC migration and early *sox10* deficits, supporting its role in neural crest development and migration to the gut [15]. This model also showed that in vivo pharmacological inhibition of *Dyrk1a* during development resulted in a significant decrease in gut motility. Gut phenotypes have yet to be assessed in zebrafish, though a *dyrk1a* mutant model has been established [130]. Future studies may attempt to evaluate ENCC migration and gut motility in this model.

### 4.5. FOXP1

Forkhead-box protein P1 (*FOXP1*) is a transcription factor coding for a transcriptional repressor protein that plays an important role in regulating neuronal development in vertebrates [131,132]. Studies in *FOXP1* haploinsufficient mice revealed significant GI dysfunction, including impaired esophageal sphincter relaxation and delayed gut transit due to reduced smooth muscle proliferation and dysregulated contractility [133]. These mice exhibited abnormal peristalsis, characterized by retrograde contractions and pendular movements. These findings point toward disrupted ENS regulation of the intestinal smooth muscle and underscore the potential role of *FOXP1* in GI sensorimotor integration. These findings could be further developed in a zebrafish model.

### 4.6. MECP2

Rett syndrome (RTT) is an X-linked NDD that primarily impacts females [134]. Patients with RTT typically experience normal physiological and neurological development leading up to 6–18 months of age, followed by a sharp regression in acquired motor skills, decline in language ability, apraxia, and growth failure [135,136]. Prevalent GI symptoms include oropharyngeal dysphagia, gastroesophageal reflux, dysmotility, and constipation [137]. *Mecp2*-knock out mice recapitulate human GI dysfunction, showing signs of significant GI hypomotility, vomiting, shortened colon length, and altered nNOS (neuronal nitric oxide synthase) expression [138,139]. Mutant zebrafish models reveal *mecp2*’s role in immunological dysregulation as reflected by an increased total neutrophil count as well as chronic inflammation of the intestines [140].

### 4.7. Neuroligin-3

*Neuroligin-3* is a synaptic adhesion molecule that has been linked to autism and GI distress [141]. A mouse model of an autism-linked variant in *neuroligin-3* R451C shows diverse gut phenotypes, including faster transit, increased numbers of myenteric neurons in the small intestine [141], increased mucous layer density in the distal ileum epithelium with an associated change in microbial distribution [142]. *Neuroligin* is expressed in both the enteric neurons and glia in mice [143] and as such these altered GI functions identified in the R451C variant could be intrinsic to altered enteric neuron function. These findings could also be further developed in a zebrafish model.

### 4.8. SHANK3

Phelan–McDermid Syndrome (PMS) is caused by mutations or deletions on chromosome 22 that include the SH and multiple ankyrin repeat domains 3 gene (*SHANK3)*. Haploinsufficiency of *SHANK3* causes symptoms including hypotonia, impaired speech, and ASD [144,145]. While *SHANK3* is well known for its role as a scaffolding protein at excitatory synapses in the CNS [146], increasing evidence shows its expression in the GI tract, thus suggesting that gut-intrinsic mechanisms may be involved in causing those symptoms [8,147]. GI symptoms such as reflux, constipation, cyclic vomiting, and feeding difficulties are also frequent among PMS individuals [148,149]. Patient-derived enterocytes show reduced expression of zinc transporters ZIP2 and ZIP4, likely due to *SHANK3* loss, which may underlie common zinc deficiency in PMS [150]. 

*Shank3B-/-* mice exhibit disrupted intestinal motility, altered barrier function, and changes in the morphology of the colon [151]. Even heterozygous mutants showed slowed transit and abnormal crypt-villus organization, alongside *Shank3* expression in enteric neurons [151]. Changes in microbiota have also been observed in *Shank3*-deficient mice, including reductions in *Lactobacillus reuteri*, whose supplementation reverses social deficits in these mice via CNX signaling [152]. In addition, *SHANK3* has been found in vagal sensory neurons that regulate systemic inflammation [153], potentially bridging gut and CNS phenotypes.

Zebrafish *shank3ab* mutants generated using CRISPR-Cas9 also showed the dysmotility phenotype observed in humans with PMS [13]. Despite normal enteric neuron numbers and intact epithelial architecture, these mutant zebrafish exhibit a significant reduction in 5-HT-producing EECs, which are essential for coordinating motility [13]. Transcriptomic data confirmed *shank3* expression in these cells, supporting EEC dysfunction as a possible cause of the impaired motility [13]. These findings may help explain lower peripheral 5-HT levels reported in PMS patients, differing from the frequently elevated levels seen in idiopathic ASD [148]. 

### 4.9. TCF4

Inheriting an autosomal dominant mutation of the Transcription Factor 4 (*TCF4*) gene significantly impacts neurodevelopment in ways that produce a clinical condition known as Pitt–Hopkins syndrome that has significant overlap with ASD [154,155,156]. TCF4 mutations are also linked with GI symptoms including constipation and gastroesophageal reflux [157,158].

Mouse TCF4 models reveal reduced motility are impacted more significantly in the upper intestine and lower distal colon [159]. Homozygous mutations in zebrafish cause stunted growth after three weeks post fertilization with less than 1% reaching adulthood [154,160]. Additionally, zebrafish models show significant epithelial proliferation defects, with the middle and distal intestines lacking folds as well as the cell proliferation typically seen at their bases [160].

### 4.10. CHD2, SYNGAP1, SCN2A

A high-throughput study in *Xenopus tropicalis* also used CRISPR-cas9 to knock down each of the following three genes: *chd2* (*chromodomain helicase DNA-binding protein 2*), *syngap1* (*synaptic Ras GTPase activating protein*), and *scn2a* (*Type II Sodium Channel*) [15]. These genes were selected from databases compiled by Simon’s Searchlight and Ciitizen of participants who reported GI distress [161]. This data also implicated delayed ENCC migration as a convergent phenotype that could underlie the GI symptoms experienced in people with these gene variants [15,161]. This warrants further exploration in stable mouse and zebrafish mutant models and highlights a potential target for broad spectrum therapeutic intervention.

## 5. The Search for Therapeutic Treatments

Zebrafish larvae present a powerful and scalable model for testing the effects of putative therapeutics on digestive function. Their optical transparency, genetic tractability, and compatibility with high-throughput systems allow investigators to screen drug compounds that modulate gut physiology within generated models of NDDs. The effects of these compounds can be measured through a combination of behavioral and image-based digestive transit assays [162,163].

Large scale drug screening is facilitated by high-throughput behavioral tracking systems, such as the Noldus DanioVision or the ViewPoint Zebrabox platform. These enable automated quantification of larval activity in multi-well formats. Recording locomotor responses to various stimuli under controlled conditions and drug exposures can identify putative therapeutics [164,165,166,167]. Hypothetically, changes in locomotor activity can serve as indirect indicators of gut–brain axis function, as GI discomfort or satiety can influence movement patterns in zebrafish larvae [168].

Putative therapeutics can also be explored through high-content imaging pipelines, including calcium imaging of intestinal neurons or smooth muscle using *GCaMP*- and/or *CaMPARI*-expressing lines [46,49]. Applying this approach to drug screening enables targeted evaluation of candidate compounds for their ability to restore normal gut activity in ASD/NDD models. Zebrafish also support direct visualization of live digestive function through ingestion of fluorescently labeled food particles, bacteria, or paramecia [13,96,161]. These feeding assays allow real-time imaging of gut transit and have been standardized to quantify digestive function in a reproducible way. Several labs have also adapted this approach to a high throughput format using microplate readers or infrared macroscopes to measure fluorescence over time and detect evacuation events in 96-well plates [109,161,169].

Many of the molecular targets relevant to gut–brain axis dysfunction are expressed both in the central nervous system and in the gastrointestinal tract. This overlap poses both an opportunity and a challenge for therapeutic development. There is growing interest in compounds that selectively modulate digestive targets—such as enteric neurons, EECs, or smooth muscle—without influencing brain function directly. For example, dopamine D2 receptor antagonists which do not cross the blood–brain barrier, such as Domperidone, have shown clinical value in targeting peripheral symptoms such as nausea and delayed gastric-emptying without central side effects [170,171]. At present, however, much of the data surrounding these compounds is empirical. In the absence of widely accepted treatments for GI symptoms in NDDs, many families pursue trial-and-error approaches with putative medications. Zebrafish offer an opportunity to accelerate this search by enabling rapid, scalable screening of candidate compounds for their effects on ASD/NDD-relevant phenotypes, including digestive physiology.

## 6. Conclusions and Future Directions

In this review, we have highlighted the development of functional sensorimotor circuits along the zebrafish gastrointestinal tract—from the pharynx to the lower intestine—and how integration across these circuits supports swallowing, mixing, absorption, motility, and evacuation. We emphasize the ways in which zebrafish are an excellent model for understanding these conserved aspects of gut physiology and how these can be disrupted by mutations in genes linked to ASD/NDDs.

This is an emerging field of study and many open questions remain. How do different sensory modalities—mechanical, chemical, microbial—converge on common motor outputs? What is the role of neuroepithelial synapses in circuit development and plasticity? How are intrinsic and extrinsic neural pathways integrated across gut segments to produce coordinated behavior? In the context of ASD/NDDs, what is the biological basis of GI symptoms that are common in these conditions?

Addressing these questions will require continued development of genetic tools, cell-type-specific reporters, and high-resolution imaging platforms. For example, recently developed tools like trans-Tango could allow for the mapping of connectivity from EECs to enteric neurons and vagus afferents [172]. Single-cell transcriptomic datasets and emerging spatial tools will enable finer dissection of regional heterogeneity and lineage relationships [53,70,102,173]. Such datasets do not yet exist for several important gut regulators, such as DRG and ICC, among others. The development of these datasets would complement in vivo tools and enhance zebrafish as a model. All together, by charting how gut sensorimotor circuits assemble and interact, zebrafish models promise to deepen our understanding of vertebrate physiology and provide translational insight into human disorders that bridge the functions of the brain and the gut.

## Data Availability

No new data was created or analyzed in this review. Data sharing is not applicable to this article.

## Abbreviations

The following abbreviations are used in this manuscript:

5-HT	Serotonin
ACh	Acetylcholine
ASD	Autism spectrum disorder
CCK	Cholecystokinin
CHARGE	Coloboma, heart malformation, choanal atresia, retardation, genital, ear anomalies
CHD	Chromodomain helicase DNA-binding protein
CNIX	Cranial neuron 9
CNS	Central nervous system
CNVII	Cranial neuron 7
CNX	Cranial neuron 10
CNTNAP2	Contactin-associated protein 2
DPF	Days post fertilization
DRG	Dorsal root ganglia
DYRK1A	Dual-specificity tyrosine phosphorylation-regulated kinase 1A
EEC	Enteroendocrine cell
EC	Enterochromaffin cell
ENCC	Enteric neural crest cell
ENS	Enteric nervous system
FOXP1	Forkhead-box protein P1
GI	Gastrointestinal
GTPase	Guanine Triphosphatase
HCN4	Hyperpolarization-activated cyclic nucleotide-gated channel 4
HPF	Hours post fertilization
ICCs	Interstitial cells of Cajal
IPAN	Intrinsic primary afferent neurons
LPM	Lateral-plate mesoderm
MECP2	Methyl CpG binding protein 2
NDDs	Neurodevelopment disorders
Neurod1	Neurogenic differentiation 1
Ngn3	Neurogenin 3
NO	Nitric oxide
nNOS	Neuronal nitric oxide synthase
PA	Pharyngeal Arches
PMS	Phelan–McDermid syndrome
PTHS	Pitt–Hopkins syndrome
PYY	Peptide YY
R	Rhombomere
RAS	Rat sarcoma virus
RNA	Ribonucleic Acid
RTT	Rett syndrome
SHANK3	SH3 and multiple ankyrin repeat domains 3
TCF4	Transcription factor 4
NTS	Nucleus tractus solitarius
VIP	Vasoactive intestinal peptide
